# Was the COVID-19 Pandemic a Triggering Factor for PTSD in Adults? Results From A Systematic Review

**DOI:** 10.62641/aep.v53i4.1882

**Published:** 2025-08-05

**Authors:** Pierluigi Catapano, Matteo Di Vincenzo, Salvatore Cipolla, Roberta Murolo, Alessandra Cirino, Alessia Boiano, Beatrice Prota, Sandra Cavaliere, Antonio Volpicelli, Bianca Della Rocca, Mario Luciano, Andrea Fiorillo, Gaia Sampogna

**Affiliations:** ^1^Department of Psychiatry, University of Campania “Luigi Vanvitelli”, 80138 Naples, Italy

**Keywords:** PTSD, COVID-19, severe acute respiratory syndrome coronavirus 2 (SARS-CoV-2), mental health, risk factors

## Abstract

**Background:**

The COVID-19 pandemic has represented a traumatic event for the general population, being associated with significant levels of uncertainty for the future, anxiety and depressive symptoms, especially in the first months of the health crisis. The adoption of strict containment measures, lockdown and interruption of all unnecessary activities have had a significant impact on the mental health of the general population. Moreover, the COVID-19 pandemic has been considered a very stressful event (which could be defined as “traumatic”), being associated with significant morbidity and mortality and being completely unpredictable. Based on such premises, we conducted a systematic review of the available literature in order to identify all studies providing epidemiological data and statistics on the prevalence and characteristics of post-traumatic stress disorder (PTSD) in the general population during the COVID-19 pandemic.

**Methods:**

An extensive literature search has been conducted across PubMed, Scopus, and Web of Science from the inception of each database until 15 November 2024.

**Results:**

Forty-one papers have been included in the review; the majority of the studies have been conducted in Italy and China. A significant heterogeneity in prevalence rates, ranging from 0.5% to 70.2%, and psychometric tool used was found. The most common risk factors for developing PTSD in the framework of the COVID-19 pandemic included: female gender, social isolation, impact on daily routine. The most relevant protective factor includes older age.

**Conclusions:**

Future research should aim to standardize assessment tools and criteria to enhance the comparability and reliability of findings in the field of trauma-related research studies.

## Introduction

The COVID-19 pandemic was officially declared by the World Health Organization on 11 March 2020, following the global outbreak of severe acute respiratory syndrome coronavirus 2 (SARS-CoV-2) [1]. At the end of the emergency on 3 May 2023, almost 7 million deaths due to the disease had been counted around the world [2], along with negative psychosocial consequences [3].

Mental health problems related to COVID-19 pandemic have been extensively studied among populations most exposed to contagion. Infected patients, suspected cases, quarantined people [4], as well as COVID-19 survivors [5, 6, 7], were at higher risk of developing depressive and anxious symptoms. First-line healthcare professionals involved in high-risk and strenuous work routine reported higher rates of burn-out, mental exhaustion, depressive and anxiety symptoms, as well as disrupted sleep [8, 9, 10].

Nevertheless, the impact of COVID-19 pandemic on mental health was not limited to special groups of subjects. Several emotional challenges, such as fear of infection and contagion [11, 12], uncertainty related to the unavailability of effective therapeutic strategies, as well as unexpected losses [13], were commonly experienced by the general population, especially during the first months of the COVID-19 pandemic. Furthermore, the strict containment measures adopted by most national governments resulted in disruption of daily routines, social isolation as well as financial concerns due to the interruption of economic activities [14, 15, 16]. The COVID-19 pandemic has been a very challenging event for the mental health of the general population. The pandemic experience has been very heterogeneous. For example, for people who experienced the loss of a loved one or lived a life-threatening condition due to the infection, pandemic can be referred as a “traumatic event”, while for others, it has been only associated with high levels of stress.

General population reported high levels of acute stress, distress and post-traumatic stress disorder (PTSD) as a consequence of COVID-19 pandemic [17, 18, 19]. PTSD includes intrusive symptoms (e.g., distressing memories, dreams, flashbacks), avoidant behaviors, negative alterations in cognition, mood and arousal, as result of direct or indirect exposure to a traumatic event [20]. Overall, PTSD prevalence had been estimated to be 5–10% in the general population, being higher among women [21]. However, the traumatic impact of the COVID-19 pandemic may have reasonably increased the rate of the disorder.

Based on such premises, we performed a systematic review in order to: (1) provide updated information on the prevalence rates of PTSD in the general population following the COVID-19 pandemic; (2) assess the most common assessment tools used to formulate the PTSD diagnosis; and (3) identify the predictive and the protective factors of PTSD in the general population.

## Data and Methods

### Search Strategy 

This systematic review has been realized following a multi-step procedure, including: (1) definition of the research question; (2) searching literature; (3) data extraction and data synthesis; (4) presentation of results. The following outcomes have been considered: prevalence of PTSD in the adult general population and assessment tools used to evaluate PTSD. Predictors and protective factors were also collected when available.

The following keywords: “(TITLE-ABS-KEY ((covid-19) OR (sars-cov-2)) AND TITLE-ABS-KEY (((ptsd) OR (post AND traumatic AND stress AND disorder))) AND TITLE-ABS-KEY ((adult*)))” were entered into PubMed. On Scopus, research was performed using the following term: “(TITLE-ABS-KEY ((covid-19) OR (sars-cov-2)) AND TITLE-ABS-KEY (((ptsd) OR (post AND traumatic AND stress AND disorder))) AND TITLE-ABS-KEY ((adult*)))”. On Web of Science, “(covid-19 OR sars-cov-2) AND ((ptsd) OR (post AND traumatic AND stress AND disorder)) AND (adult*)” were used as keywords. Database searches were conducted from the inception of each source to 15 November 2024.

**Fig. 1.**
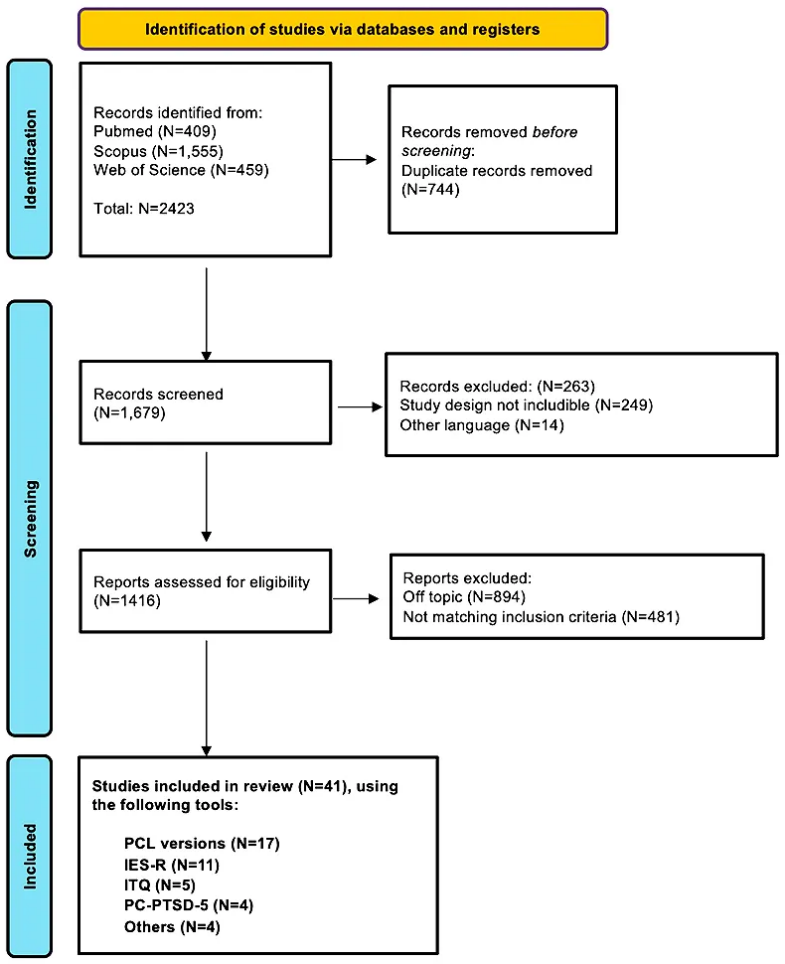
**Flowchart of the included studies**. IES-R, Impact of Event Scale-Revised; ITQ, International Trauma Questionnaire; PCL, Posttraumatic Stress Disorder Checklist; PC-PTSD-5, Primary Care PTSD Screen for DSM-5; PTSD, post-traumatic stress disorder.

The Preferred Reporting Items for Systematic Reviews and Meta-Analyses (PRISMA) guidelines have been adopted [22]. The full PRISMA checklist can be found in “**Supplementary file 1**”. A PRISMA flowchart has been included (Fig. 1). The ZOTERO free software (version 6.0.36) has been used for managing references and for deleting duplicates.

### Selection Criteria 

Studies were eligible for inclusion if they: (a) included adults (18 years or above) from the general population; (b) used validated tools to assess the prevalence and severity of PTSD; (c) were written in English; (d) referred to COVID-19 pandemic period (30 January 2020–5 May 2023). Studies were excluded if they: (a) included any kind of special population; (b) were clinical trials, randomized controlled trials, reviews, meta-analyses, study protocols, case reports, comments, letters to editor, expert opinions, or qualitative studies.

### Selection Process and Data Extraction

Nine reviewers (AB, AC, BP, MDV, PC, SCa, SCi, AV, RM) independently assessed studies obtained from the database searches in three phases: search of literature, title–abstract screening, and full-text screening, and then synthesized them in a tabular format. A senior researcher (GS) was consulted if needed. For each included study, the following data were collected: authors, country and year of publication, sample size, assessment tools, PTSD prevalence, predictors and protective factors of PTSD. The authors screened the articles and then performed a full-text review of those articles included by titles and abstracts. Disagreements among reviewers were resolved through discussion and with the assistance of a senior researcher (GS). The senior author (AF) reviewed the complete study methodology and provided comments to improve papers’ extraction.

### Risk of Bias Assessment

Three authors (PC, MDV and SCi) with extensive experience in risk of bias evaluation conducted independent assessments of the risk of bias for each selected study utilizing the ROBINS-E tool, a systematic approach designed for evaluating bias in observational research [23]. Any disagreements were resolved through discussions with senior researchers (AF and GS) when necessary. The overall risk of bias was evaluated as high.

## Results

Based on the search strategy, 2423 papers were identified. Seven-hundred and forty-four were duplicates and were removed. Therefore, 1679 papers were evaluated in title and abstract, and following the screening procedure, N = 1416 papers were analyzed in full-text. Finally, 41 papers have been included in the review. The majority of the studies have been conducted in Italy and China.

### Prevalence Rates of PTSD During the COVID-19 Pandemic

The prevalence rates of PTSD varied among the studies, ranging from a minimum of 0.5% in the Lueger-Schuster *et al*. (2022) [24] study in Austria (at the second measurement—from 14 January 2021 to 29 March 2021—since this study evaluated prevalence of PTSD during four different moments of the COVID-19 pandemic) to a maximum of 70.16% in the study by Passavanti *et al*. (2021) [25], carried out in different countries. The lowest prevalence was observed using the Posttraumatic Stress Disorder Checklist (PCL), whereas the highest prevalence was recorded with the use of the Impact of Event Scale-Revised (IES-R).

The overall sample size of included participants ranged from 84 persons in the study by Gill *et al*. (2022) [26] to 31,557 in the research performed by Elhadi *et al*. (2022) [27].

### Assessment Tools Used in the Included Studies

The most frequent assessment tools adopted to identify the presence of PTSD included versions of the Posttraumatic Stress Disorder Checklist (PCL) [28, 29, 30, 31], the Impact of Events Scale-Revised (IES-R) [32], the International Trauma Questionnaire (ITQ) [33], and the Primary Care PTSD Screen for DSM-5 (PC-PTSD-5) [34]. Further instruments [35, 36, 37, 38] were also used in a small number of studies (Table 1, Ref. [24, 25, 26, 27, 38, 39, 40, 41, 42, 43, 44, 45, 46, 47, 48, 49, 50, 51, 52, 53, 54, 55, 56, 57, 58, 59, 60, 61, 62, 63, 64, 65, 66, 67, 68, 69, 70, 71, 72, 73, 74]).

**Table 1.** **Assessment tools for PTSD used in the included studies**.

Assessment tool	Related studies
Posttraumatic Stress Disorder Checklist (PCL) versions	
20-item Post-Traumatic Stress Disorder Checklist for Diagnostic and Statistical Manual of Mental Disorders, 5th Edition (DSM-5)	Chung *et al*. (2022) [39]; Sujan *et al*. (2023) [40]; Alleaume *et al*. (2022) [41]; Samuelson *et al*. (2022) [42]; Ikizer *et al*. (2021) [43]; Shen *et al*. (2021) [44]; Guo *et al*. (2021) [45]; Casagrande *et al*. (2020) [46]; Liu C *et al*. (2021) [47]; Liu N *et al*. (2020) [48]; Sherman *et al*. (2020) [49]
17-items Post-Traumatic Stress Disorder Checklist for civilians, based on DSM-IV criteria	Wang *et al*. (2022) [50]; Liu *et al*. (2022) [51]; Nzimande *et al*. (2022) [52]
17-items Post-Traumatic Stress Disorder Checklist Survey, based on DSM-5 criteria	Alatawi *et al*. (2020) [53]; Alshehri *et al*. (2020) [54]
4-item Post-Traumatic Stress Disorder Checklist for DSM-5	Abdalla *et al*. (2021) [55]
Impact of Event Scale-Revised (IES-R)	El Khoury-Malhame *et al*. (2023) [57]; El Khoury-Malhame *et al*. (2023) [56]; Elhadi *et al*. (2022) [27]; Aljaberi *et al*. (2022) [58]; Scuri *et al*. (2022) [59]; Karaivazoglou *et al*. (2021) [60]; Mukherjee *et al*. (2021) [61]; Passavanti *et al*. (2021) [25]; Di Giuseppe *et al*. (2020) [62]; Fekih-Romdhane *et al*. (2020) [63]; Forte *et al*. (2020) [64]
International Trauma Questionnaire (ITQ)	Shevlin *et al*. (2020) [65]; Makhashvili *et al*. (2020) [66]; McGinty *et al*. (2024) [67]; Daly *et al*. (2021) [68]; Greenblatt-Kimron *et al*. (2023) [69]
Primary Care PTSD Screen for DSM-5 (PC-PTSD-5)	Généreux *et al*. (2022) [70]; Lovik *et al*. (2023) [71]; Lotzin *et al*. (2022) [72]; Lueger-Schuster *et al*. (2022) [24]
Screen for Posttraumatic Stress Symptoms (SPTSS)	Kakaje *et al*. (2021) [73]
Adult Psychiatric Morbidity Survey	Gill *et al*. (2022) [26]
Global Psychotrauma Screen for Post-Traumatic Stress Symptoms (GPS-PTSS)	Rossi *et al*. (2020) [74]
COVID-19-PTSD Questionnaire (readjusted from the PCL-5)	Forte *et al*. (2020) [38]

### Studies Adopting Different Versions of the Post-Traumatic Stress Disorder Checklist (PCL)

In 41.5% of the included studies (N = 17), the PCL scale has been used as the main assessment tool for evaluating the presence of PTSD. Different versions of the scale are available. In particular, 11 studies [39, 40, 41, 42, 43, 44, 45, 46, 47, 48, 49] used the 20-item Post-Traumatic Stress Disorder Checklist for DSM-5 (PCL-5), with a cut-off of 31 for provisional PTSD and of 33 for PTSD; two studies [50, 51] adopted the 17-items Post-Traumatic Stress Disorder Checklist for civilians, based on DSM-IV criteria (PCL-C), with a cut-off of ≥38, and one additional study [52] used the same tool, but with a threshold of ≥44; two studies [53, 54] used the 17-items Post-Traumatic Stress Disorder Checklist Survey, based on DSM-5 criteria (PCL-S) with a cut-off of ≥44, while one study [55] used the 4-item Post-Traumatic Stress Disorder Checklist for DSM-5, using a cut-off of ≥3.

Out of 17 studies using PCL versions, reliability data were available in nine studies, with satisfying level of Cronbach’s alpha values, ranging from 0.93 to 0.97 (Table 2, Ref. [39, 40, 41, 42, 43, 44, 45, 46, 47, 48, 49, 50, 51, 52, 53, 54, 55]).

**Table 2.** **Studies using PCL versions to assess PTSD**.

Author (year)	Population	PTSD prevalence	Assessment tool (cut-off)	Associated factors/Predictors/Risks factors	Protective factors
Country	(observation period)		Internal consistency		
Sujan MSH, *et al*. (2023) [40]	N = 326	Cut-off of 31: 40.5%; cut-off of 32: 37.7%; cut-off of 33: 35.9%	PCL-5 (31–33) Cronbach’s α = 0.93	Regression analysis	Regression analysis
	Male: 69%			Age ≥40 years (β = 0.23, *p * < 0.001);	Social support (family members, friends, relatives: β = –0.19, *p* = 0.002; work colleagues: β = –0.16, *p* = 0.014)
Bangladesh	Age 18–76 (mean age: 37.97 ± 13.02)			lower socio-economic status (β = 0.14, *p* = 0.021);	
				sleeping more than 9 h per day (β = 0.10, *p* = 0.046);	
	(September 2020–January 2021)			social support (healthcare providers: β = 0.17, *p* = 0.005)	
Alleaume C, *et al*. (2022) [41] France	N = 1736	17.5%	PCL-5 (≥33)	Regression analysis	Regression analysis
	Male: 826 (47.58%)		N/A	Media consumption of COVID-19 pandemic-related information more than 4 hours per day (RR = 1.53, *p* < 0.001);	Media consumption of COVID-19 pandemic related information less than 1 hour per day (RR = 0.67, *p* < 0.01)
	Age ≥18				
	(May 2020 + 1-month follow-up)				
				mild to severe anxiety during lockdown assessed by GAD-7 (RR = 3.26, *p * < 0.01);	
				COVID-19 infection (RR = 1.43, *p * < 0.001);	
				mild to severe anxiety at 1-month follow-up assessed by GAD-7 (RR = 3.02, *p * < 0.001);	
				mild to severe depression at 1-month follow-up assessed by PHQ-9 (RR = 2.44, *p * < 0.001);	
				severe sleep problems at 1-month follow-up assessed by ad hoc question (RR = 1.51, *p * < 0.001)	
Chung MC, *et al*. (2022) [39]	N = 1089	Partial-PTSD: 68.7%; full-PTSD: 12.7%	PCL-5	N/A	N/A
	Male: 382 (35%)		Cronbach’s α = 0.94; test-retest reliability = 0.82		
China	Age ≥18 (mean age: 26.36 ± 8.58)				
	(April 2020)				
Liu Y, *et al*. (2022) [51]	N = 2067	368 (17.8%)	PCL-C (≥38)	Regression analysis	N/A
	Male: 469 (22.7%)		Cronbach’s α = 0.96	Being male (β = –0.07, *t* = –3.47, *p* = 0.001);	
China	Age ≥18			Being part of ethnic minorities (β = 0.05, *t* = 2.58, *p* = 0.01);	
	(March 2020)				
				High personal monthly income (β = 0.09, *t* = 4.4, *p * < 0.001);	
				Being exposed to Wuhan (β = –0.11, *t* = –5.06, *p * < 0.001);	
				Contact with COVID-19 patients (β = –0.13, *t* = –6.02, *p * < 0.001);	
				Isolation (β = –0.06, *t* = –2.85, *p* = 0.004);	
				Experience of seeing a doctor during pandemic (β = 0.10, *t*= –4.79, *p * < 0.001);	
				Lower self-efficacy assessed by GSES (β = –0.08, *t* = –3.68, *p * < 0.001);	
				Lack of social support assessed by PSSS (β = –0.15, *t* = –6.54, *p * < 0.001);	
				Negative coping styles assessed by SCS (β = –0.20, *t* = –9.42, *p * < 0.001)	
Nzimande NP, *et al*. (2022) [52]	N = 498	173 (35.4%)	PCL-C (≥44)	Association in bivariate analysis	Regression analysis
	Male: 179 (36.6%)		Cronbach’s α = 0.939	Being unemployed (*p* = 0.048),	Older age (OR = 0.97, 95% CI: 0.95∼0.99, *p* = 0.04); Feeling to have enough emotional support from family and relatives (OR = 0.27, 95% CI: 0.14∼0.53, *p * < 0.001)
South Africa	Age ≥18 (mean age: 30.8 ± 9.5)			being female (*p * < 0.001),	
				feeling social isolation (*p * < 0.001),	
	(June–December 2020)			reporting COVID-19 has negative impact on daily life (*p* = 0.005);	
				reporting less emotional support from family and friends (*p* < 0.001)	
				Regression analysis	
				Being female (OR = 2.19, 95% CI: 1.41∼3.39, *p * < 0.001);	
				Feeling more socially isolated (OR = 1.17, 95% CI: 1.08∼1.27, *p * < 0.001)	
Samuelson KW, *et al*. (2022) [42]	N = 467	Probable PTSD: 22.5%	PCL-5 (≥31 for probable PTSD)	Supervised machine learning (Random Forest, XGBoost, SVM-RBF, Elastic Net)	N/A
	Male: 31.5%		N/A		
	Mean age 33.14 ± 12.95			COVID-19 pandemic coping self-efficacy (lower levels associated with higher PTSD);	
USA					
	(May–July 2020)			Forward-focused coping (lower levels associated with higher PTSD)	
Wang J, *et al*. (2022) [50]	N = 1150	26 (2.3%)	PCL-C	Structural equation modeling	N/A
	Male: 410 (35.7%)		Composite reliability = 0.910	COVID-19 pandemic information overload (β = 0.190, *p * < 0.001);	
China	Mean age: 37.7 ± 13.91				
				Depression (β = 0.757, *p * < 0.001)	
	(July 2020–March 2021)				
Abdalla S, *et al*. (2021) [55]	N = 1450	21.7%	Four-item PCL (≥3)	Regression analysis	Regression analysis
	Male: 725 (48.2%)		N/A	Being female (OR = 1.5, 95% CI: 1.1∼2.1, *p* = 0.024);	Age >60 years (OR = 0.6, 95% CI: 0.3∼1.0, *p* = 0.05)
USA	Age ≥18				
	(March–April 2020)			high COVID-19-related stressor score (OR = 3.3, 95% CI: 2.1∼5.1, *p * < 0.0001)	
Guo X, *et al*. (2021) [45]	N = 1009	57 (5.6%)	PCL-5 (>33)	Hierarchical multivariate regression analysis	N/A
	Male: 359 (35.6%)		N/A	Subjective fear (β = 0.504, *p * < 0.001);	
China	Median age 38.3 ± 11.5			Currently in Hubei (β = 0.091, *p * < 0.05);	
				High-risk public (β = 0.056, *p * < 0.05)	
	(January–February 2020)				
Ikizer G, *et al*. (2021) [43]	N = 685	328 (47.9%)	PCL-5 (scores ≥31 indicate provisional PTSD); PTGI (higher scores indicate higher levels of PTG and PTD)	Regression analysis (PTS)	N/A
	Male: 237 (34.6%)			Young age (β = –0.18, *t* = –3.66, *p * < 0.001);	
Turkey	Mean age 34.63 ± 15.04			lower education level (β = –0.12, *t* = –2.69, *p * < 0.01);	
				being single (β = –0.09, *t* = –2.01, *p * < 0.05);	
	(June–August 2020)		Cronbach’s α = 0.95	longer time spent on social media following COVID-19 pandemic related news (β = 0.12, *t* = 3.57, *p * < 0.001);	
				time spent home due the COVID-19 pandemic (β = 0.09, *t* = 2.37, *p * < 0.05);	
				perceived health risk of the disease (β = 0.19, *t* = 4.83, *p * < 0.001);	
				experience of financial loss (β = –0.10, *t* = –2.81, *p * < 0.01);	
				perceived financial risk (β = 0.09, *t* = 2.2, *p * < 0.05);	
				perceived stress assessed by PSS (β = 0.43, *t* = 12.93, *p * < 0.001);	
				intrusive rumination (β = 0.45, *t* = 13.53, *p * < 0.001) and deliberate rumination (β = 0.15, *t* = 4.6, *p * < 0.001) assessed by ERRI	
				Regression analysis (PTG)	
				Lower education level (β = –0.10, *t* = 2.06, *p * < 0.05);	
				perceived financial risk (β = 0.12, *t* = 2.73, *p * < 0.01);	
				deliberated rumination assessed by ERRI (β = 0.42, *t* = 9.31, *p * < 0.001)	
				Regression analysis (PTD)	
				Younge age (β = –0.20, *t* = 4.08, *p * < 0.001); lower education level (β = –0.13, *t* = –2.96, *p * < 0.01);	
				being single (β = –0.17, *t* = –3.76, *p * < 0.001);	
				perceived health risk of the disease (β = 0.10, *t* = 2.41, *p * < 0.05);	
				Perceived financial risk (β = 0.12, *t* = 2.85, *p * < 0.01);	
				Perceived stress assessed by PSS (β = 0.43, *t* = 2.79, *p * < 0.001); Intrusive rumination (β = 0.12, *t* = 2.79, *p * < 0.01) and deliberate rumination (β = 0.19, *t* = 4.67, *p * < 0.001) assessed by ERRI	
				Regression analysis (PLC-5 ≥31)	
				Young age (OR = 0.98, *p* = 0.003);	
				lower education level (OR = 0.85, *p* = 0.026);	
				longer time spent on social media (OR = 1.003, *p* = 0.03);	
				Perceived health risk (OR = 1.68, *p * < 0.001);	
				perceived stress assessed by PSS (OR = 1.18, *p * < 0.001); intrusive (OR = 3.15, *p * < 0.001) and deliberate rumination (OR = 1.92, *p * < 0.001) assessed by ERRI	
Liu C, *et al*. (2021) [47]	N = 2858	558 (19.5%)	PCL-5 (N/A)	Regression analysis	Regression analysis
	Male: 46.4%		Cronbach’s α = 0.97	Being male (OR = 1.824, 95% CI: 1.477∼2.251, *p * < 0.001);	Living or traveling in Wuhan (OR = 0.694, 95% CI: 0.501∼0.961, *p* < 0.05);
China	Age ≥18				
	(February 2020)			age between 26 and 30 years (OR = 1.796, 95% CI: 1.103∼2.925, *p * < 0.05);	
					Sporadic media exposure (OR = 0.768, 95% CI: 0.601∼0.981, *p * < 0.05)
				lower education (being undergraduate: OR = 1.679, 95% CI: 1.193∼2.363, *p * < 0.01; junior college education: OR = 1.94, 95% CI: 1.305∼2.885, *p * < 0.01;	
				high school or technical school education: OR = 2.373, 95% CI: 1.573∼3.581, *p * < 0.01);	
				being married (OR = 1.368, 95% CI: 1.022∼1.831, *p * < 0.01);	
				nonprofessional employees (OR = 1.721, 95% CI: 1.129∼2.621, *p * < 0.05);	
				direct exposure to COVID-19 (OR = 1.186, 95% CI: 1.069∼1.315, *p * < 0.01);	
				negative impact on livelihood (some impact: OR = 1.499, 95% CI: 1.123∼1.999, *p * < 0.01; relatively large impact: OR = 3.054, 95% CI: 2.275∼4.101, *p * < 0.001; very large impact: OR = 2.590, 95% CI: 1.879∼3.571, *p * < 0.001);	
				psychological problems (OR = 2.026, 95% CI: 1.609∼2.552, *p * < 0.001);	
				having 2-weeks illness (OR = 1.554, 95% CI: 1.074∼2.248, *p * < 0.05).	
				Regression analysis for the combined effect of gender and age on PTS symptoms shows that	
				men aged 18–50 may experience a high degree of PTS symptoms, compared with females aged 18–25 years old (Male 18–25: OR = 2.647, 95% CI: 1.711∼4.097, *p * < 0.001; male 26–30: OR = 2.864, 95% CI: 1.725∼4.695, *p * < 0.001; male 31–40: OR = 1.962, 95% CI: 1.181∼3.259, *p * < 0.01; male 41–50: OR = 1.880, 95% CI: 1.050∼3.364, *p * < 0.05)	
Shen X, *et al*. (2021) [44]	N = 2361	219 (9.28%)	PCL-5 (>33)	Regression analysis	Regression analysis
	Male: 942 (39.9%)		Cronbach’s α = 0.962	Female sex (β = 0.038, 95% CI: 0.006∼1.947, *p* = 0.046);	Age >60 years (β = –0.063, 95% CI: –5.278∼–1.245, *p* = 0.001);
China	Age: 18–77 (mean age: 29.72 ± 6.94) (February 2021)				
				relative or friend with COVID-19 (β = 0.041, 95% CI: 0.122∼3.528, *p* = 0.036);	being married (β = –0.097, 95% CI: –3.955∼–1.209, *p * < 0.001);
				poor health (β = 0.184, 95% CI: 0.379∼9.354, *p* = 0.034)	agreement that information about COVID-19 has been released in a timely manner (β = –0.347, 95% CI: –10.893∼–8.713, *p * < 0.001));
					perception that COVID-19 pandemic had a limited impact on their life (β = –0.069, 95% CI: –3.394∼–0.712, *p* = 0.003);
					agreement that the local prevention initiatives were sophisticated (β = –0.165, 95% CI: –9.533∼–0.168, *p* = 0.042)
Alatawi Y, *et al*. (2020) [53]	N = 1249	22.66%	PCL-S (≥45)	Regression analysis (Method 3 – combination of cut-off ≥45 and DSM criteria)	Regression analysis
	Male: 620 (49.64%) Age ≥18 (June 2021)		N/A		High level of health literacy (OR = 0.97, 95% CI: 0.95∼0.99, *p * < 0.001)
Saudi Arabia				High level of perceived threat assessed by BIP-Q5 (OR = 1.17, 95% CI: 1.13∼1.19, *p * < 0.001);	
				history of mental illness (OR = 4.20, 95% CI: 1.93∼9.15, *p * < 0.001);	
				being divorced/widowed (OR = 2.83, 95% CI: 1.12∼7.17, *p * < 0.05);	
				being married (OR = 1.55, 95% CI: 1.07∼2.25, *p * < 0.05)	
Alshehri FS, *et al*. (2020) [54]	N = 1374	Cut-off: 22.63%; PTSD criteria: 24.8%; PTSD combined: 19.6%	PCL-S (three methods: cut-off ≥45; PTSD criteria; PTSD combined)	Stepwise multivariable logistic regression analysis	Stepwise multivariable logistic regression analysis
	Male: 674 (49.05%)			Female gender (OR = 1.37, *p * < 0.05);	
Saudi Arabia	Age ≥18			Confirmed or suspected COVID-19 infection (OR = 1.89, *p * < 0.05);	High resilience (OR = 0.58, *p * < 0.05)
	(June 2020)		N/A		
				Single marital status (OR = 1.45, *p * < 0.05);	
				Family death due to COVID-19 (OR = 1.68, *p * < 0.05); Previous psychiatric condition (OR = 2.67, *p * < 0.05)	
Casagrande M, *et al*. (2020) [46]	N = 2291	173 (7.6%)	Modified version of PCL-5 (>1.5 SD from the mean score) Cronbach’s α = 0.94	Regression analysis	
	Male: 580 (25.3%) Age 18–89 (mean age: 30 ± 11.5) (March–April 2020)			Lower sleep quality measured by PSQI global score (β = 0.14, 95% CI: 0.5∼0.76, *p * < 0.001);	
Italy				greater generalized anxiety symptomatology assessed by GAD-7 global score (β = 0.41, 95% CI: 1.14∼1.37, *p * < 0.001);	
				higher psychological distress assessed by PGWΒ global score (β = –0.36, 95% CI: –0.41∼–0.33, *p * < 0.001)	
Liu N, *et al*. (2020) [48]	N = 285	20 (7%)	PCL-5 (≥33)	Regression analysis (model 3, R^2^ = 0.303)	N/A
	Male: 130 (45.6%)		N/A	Being female (β = 0.102, *t* = 1.958, *p * < 0.05);	
China	Age >18 (January–February 2020)			bad or very bad subjective sleep quality (β = 0.312, *t* = 4.816, *p * < 0.001);	
				being unable to fall asleep (β = 0.172, *t* = 2.750, *p * < 0.01)	
Sherman AC, *et al*. (2020) [49]	N = 591	29 (5.38%)	PCL-5 (>33)	Associations in bivariate analyses	N/A
	Male: 133 (22.5%)		N/A	Prior mental health history (*p * ≤ 0.0001);	
USA	Age ≥18 (mean age: 51.19) Only 544 completed PCL-5 (May–June 2020)			greater disruption in daily life (difficulties caring for others, arranging childcare, sustaining activities or religious pursuits, maintaining connection with family and friends) (*p * ≤ 0.0002);	
				perceived SARS-CoV-2 infection (*p * ≤ 0.0005);	
				adverse changes in employment (*p * ≤ 0.0005);	
				more stringent efforts to shelter at home (*p * ≤ 0.0008)	
				Regression analyses	
				Prior mental health history (OR = 6.44, 95% CI: 2.10∼19.72, *p * < 0.001);	
				increased disruption in daily life (OR = 1.20, 95% CI: 1.09∼1.31, *p * < 0.0002)	

COVID-19, Coronavirus disease-19; ERRI, Event Related Rumination Inventory; GAD-7, General Anxiety Disorder questionnaire; GSES, General Self-Efficacy Scale; N/A, Not addressed; PCL-5, The 20-item Post-Traumatic Stress Disorder Checklist for DSM-5; PCL-C, The 17-items Post-Traumatic Stress Disorder Checklist for civilians, based on DSM-IV criteria; PCL-S, 17-items Post-Traumatic Stress Disorder Checklist Survey, based on DSM-5 criteria; PGWB, Psychological General Well-Being questionnaire; PHQ-9, Patient Health Questionnaire-9; PSQI, Pittsburg Sleep Quality Index; PSS, Perceived Stress Scale; PSSS, Perceived Social Support Scale; PTD, Post-traumatic depreciation; PTG, Post-traumatic Growth; PTGI, Post-traumatic Growth Inventory; PTS, Post-traumatic stress; SARS-CoV-2, severe acute respiratory syndrome coronavirus 2; SCS, Self-Report Coping Scale; SD, standard deviation; OR, Odds Ratio; CI, Confidence Interval; RR, Relative Risk; BIP-Q5, 5-Item Brief Illness Perception Questionnaire.

### Studies Adopting Different Versions of the IES-R (Impact of Event Scale-Revised) 

IES-R has been used as main assessment tool for evaluating the presence of PTSD in eleven papers out of 41 (26.8%) [25, 27, 56, 57, 58, 59, 60, 61, 62, 63, 64]. A cut-off threshold >33 was established for considering the presence of PTSD. Only in the study by Aljaberi and colleagues (2022) [58] a threshold >23 was considered. The prevalence rate of PTSD in studies using IES-R ranged from 19.8% in the study by Elhadi *et al*. (2022) [27] carried out in Libya to 70.16% in the study by Passavanti *et al*. (2021) [25] carried out in multiple countries. Out of 11 studies using IES-R, reliability data were available in five studies, with a satisfying level of Cronbach’s alpha values, ranging from 0.86 to 0.95 (Table 3, Ref. [25, 27, 56, 57, 58, 59, 60, 61, 62, 63, 64]).

**Table 3.** **Studies using IES-R to assess PTSD**.

Author (year)	Population	PTSD prevalence	Assessment tool (cut-off)	Associated factors/Predictors/Risks factors	Protective factors
Country	(observation period)		Internal consistency		
El Khoury-Malhame M, *et al*. (2023) [57] Lebanon	N = 252	41%	IES-R (>33)	Linear regression	Linear regression
	Male: 71 (28.3%)		Cronbach’s α = 0.95	Higher impact of events (β = 0.13, *p * < 0.001);	More gratitude (β = 0.52, *p * < 0.001)
	Age 18–43 (mean age: 25 ± 8.25)			Knowing anyone who died from COVID-19 (β = 3.76, *p* = 0.008)	
	(March 2021)				
El Khoury-Malhame M, *et al*. (2023) [56] Lebanon	N = 348	44.5%	IES-R (>33)	Regression analyses	Regression analyses
	Male: 98 (28.16%) Lebanese adults (Age: N/A) (May–June 2020)		(named IES-22 in this study) Cronbach’s α = 0.94	Insomnia assessed by PSQI (β = 0.46, 95% CI: 1.96∼2.97, *p * < 0.001)	Being healthcare provider (β = –0.15, 95% CI: –12.46∼–3.18, *p* = 0.001);
					Higher gratitude assessed by GQ-6 (β = –0.13, 95% CI: –0.63∼–0.09)
Elhadi M, *et al*. (2022) [27] Libya	N = 31,557 Male: 10,802 (34.2%) Age: 18–80 (May 2020)	6245 (19.8%)	IES-R (≥33)	Regression analysis	N/A
			Internal consistency = 0.86	Younger age (OR = 0.995, 95% CI: 0.991∼0.998, *p* = 0.003);	
				Female gender (OR = 1.07, 95% CI: 1.005∼1.143, *p* = 0.034);	
				Being unmarried (OR = 1.139, 95% CI: 1.06∼1.22, *p * < 0.001);	
				Higher education level (OR = 1.59∼1.78, *p * < 0.01);	
				Internally displaced (OR = 1.26, 95% CI: 1.09∼1.45, *p* = 0.001);	
				Work status changes during COVID-19 pandemic (increased workload: OR = 2.07, 95% CI: 1.82∼2.36, *p * < 0.001; teleworking: OR = 1.6, 95% CI: 1.43∼1.78, *p * < 0.001; Work suspended: OR = 1.13, 95% CI: 1.04∼1.23, *p* = 0.004);	
				Infected with COVID-19 without hospitalization (OR = 3, 95% CI: 2.25∼4.004, *p * < 0.001);	
				Recent contact with infected patients (OR = 3.64, 95% CI: 2.94∼4.51, *p * < 0.001);	
				Family member or loved ones being infected with COVID-19 with (OR = 4.01, 95% CI: 3.07∼5.24, *p * < 0.001) and without hospitalization (OR = 1.64, 95% CI: 1.38∼1.94, *p * < 0.001);	
				Financial issues (OR = 1.51, 95% CI: 1.42∼1.60, *p * < 0.001); Domestic violence or abuse (OR = 2.01, 95% CI: 1.89∼2.14, *p * < 0.001);	
				Suicidal ideation during lockdown (OR = 2.49, 95% CI: 2.26∼2.74, *p * < 0.001)	
Aljaberi MA, *et al*. (2022) [58]	N = 999	360 (36%)	IES-R (>23)	N/A	N/A
	Male: 445 (45.5%)		Excellent composite reliability coefficients were above 0.70		
Malaysia	Mean age: 33.06 ± 9.3				
	(April–May 2020)				
Scuri S, *et al*. (2022) [59]	N = 480	154 (37.75%)	IES-R (>33)	N/A	N/A
	Male: 156 (38.24%)		N/A		
Italy	Age: 18–79 (mean age: 37.54 ± 14.45)				
	(March–May 2020)				
Karaivazoglou K, *et al*. (2021) [60] Greece	N = 1468	Partial: 272 (19.6%); Probable: 121 (8.7%); Definite: 506 (36.4%) Cut-off >33: 627 (45.1%)	IES-R (partial PTSD: 24–32; Probable PTSD: 33–36; Definite PTSD: ≥37)	Regression analysis	Regression analysis
	Male: 391 (27.1)			Female gender (β = 6.451, *p * < 0.001);	No history of psychiatric treatment (β = –4.028, *p * < 0.001);
	Age ≥18			COVID-19 pandemicrelated worry (slight worry: β = 6.837, *p* = 0.007; enough worry: β = 11.649, *p* < 0.001; great worry: β = 23.865, *p* < 0.001); Protective measures perceived as probably not effective (β = 12.903, *p* = 0.020)	
	(April–May 2020)		N/A		Postgraduate degree (β = –3.401, *p* = 0.003); PhD degree (β = –5.737, *p* = 0.003);
					No adherence to protective measures (β = –6.54, *p* = 0.049)
Mukherjee M, *et al*. (2021) [61] India	N = 658	Low: 62.8%; Partial: 18.4%; Moderate: 7.3%; High: 11.6% Total over cut-off >33: 18.9%	IES-R (low PTSD: 0–24; Partial PTSD: 24–32; Moderate PTSD: 33–38; High PTSD: >38)	Regression analysis	N/A
	Male: 351 (53%)			Higher media use (β = 0.35, *p * < 0.01)	
	Age 18–68				
	(March 2020)		N/A		
Passavanti M, *et al*. (2021) [25]	N = 1612 Male: 644 (40%) Mean age: 28 ± 9.36 (April 2020)	Mild: 250 (14.3%); Moderate: 136 (8.5%); Severe: 745 (46.8%)	IES-R (mild PTSD: 24–32; Moderate PTSD: 33–36; Severe PTSD: ≥37)	N/A	N/A
Australia, China, Ecuador, Iran, Italy, Norway, USA		Cut-off >33: 881 (55.3%)	N/A		
Di Giuseppe M, *et al*. (2020) [62] Italy	N = 5683	29.4%	IES-R (>33)	Regression analysis	Regression analysis
	Male: 1427 (25%) 18 years or older (13 March to 18 March 2020)		N/A	Female gender (β = 8.379, *p* < 0.001); Being close to positive cases (β = 2.165, *p* < 0.01); More days in lockdown (8–14 days: β = 1.299, *p* < 0.001; >14 days: β = 2.775, *p* < 0.001); Moved to new location due COVID-19 pandemic (N/A) Odds Ratio evaluation Female gender (OR = 2.72); Having positive cases nearby (OR = 1.44)	Older age (40–49: β = –1.144, *p * < 0.05; 50–59: β = –3.396, *p * < 0.001; ≥60: β = –7.005, *p * < 0.001);
					Not living with close relatives (living alone: β = –1.394, *p * < 0.01; living with partner: β = –1.481, *p * < 0.01); working from home (β = –1.233, *p * < 0.001)
					Odds Ratio evaluation
					Age >60 (OR = 0.48);
					Higher level of ODF assessed with DMRS-SR-30 (OR = 0.29)
Fekih-Romdhane F, *et al*. (2020) [63] Tunisia	N = 603	199 (33%)	IES-R (>33) N/A	Regression analysis	N/A
	Male: 26%			Gender (OR = 0.34, *p * < 0.001);	
	Age >18 (mean age: 29.2 ± 10.4) (April 2020)			Hearing or discussing with another person the details of a person’s illness or death due to COVID-19 (OR = 1.53, *p* = 0.035);	
				Being not able to communicate with loved ones (OR = 1.51, *p* = 0.031);	
				Difficulty obtaining personal supplies (OR = 2.63, *p* = 0.003);	
				Total time spent on news and events related to COVID-19 pandemic on media per day (OR = 0.63, *p* = 0.017);	
				Being exposed to photos or narratives or other details about burial of COVID-19 victims (OR = 1.65, *p* = 0.011)	
Forte G, *et al*. (2020) [64] Italy	N = 2291	635 (27.72%)	IES-R (>33)	Regression analysis	N/A
	Male: 580 (25.3%) Age 18–89 (mean age: 30 ± 11.5) (March 2020)		Cronbach’s α = 0.95	Female gender (OR = 2.39, 95% CI: 1.88∼3.05, *p * < 0.001);	
				Being aged 18–29 (OR = 1.71, 95% CI: 1.21∼2.41, *p * < 0.01);	
				Age between 30–49 (OR = 1.66, 95% CI: 1.14∼2.43, *p * < 0.01);	
				Probable direct contact with people infected by COVID-19 (OR = 1.32, 95% CI: 1.09∼1.59, *p * < 0.01);	
				Knowing people infected by COVID-19 (OR = 1.34, 95% CI: 1.09∼1.66, *p * < 0.05);	
				Knowing people in ICU due COVID-19 (OR = 1.45, 95% CI: 1∼2, *p * < 0.05);	
				Knowing people died for COVID-19 (OR = 1.88, 95% CI: 1.28∼2.77, *p * < 0.001)	

COVID-19, Coronavirus disease-19; DMRS-SR-30, Defense Mechanisms Rating Scales-Self-Report-30; GQ-6, Gratitude Questionnaire; IES-R, The Impact of Event Scale-Revised; ODF, overall defensive functioning; PSQI, Pittsburg Sleep Quality Index; ICU, Intensive care unit.

### Studies Adopting the International Trauma Questionnaire (ITQ)

In five papers (12.2%), the ITQ was used as main assessment tool for evaluating the presence of PTSD, with a threshold ≥2 [65, 66, 67, 68, 69]. The prevalence rate of PTSD ranged from 7.4% in the study by Greenblatt-Kimron *et al*. (2023) [69] to 22.0% in the study by Daly *et al*. (2021) [68]. Out of five studies using ITQ, reliability data were available in four studies, with satisfying level of Cronbach’s alpha values, ranging from 0.89 to 0.93. The remaining study [67] reported satisfactory internal reliability without indicating alpha value (Table 4, Ref. [65, 66, 67, 68, 69]).

**Table 4.** **Studies using ITQ to assess PTSD**.

Author (year)	Population	PTSD prevalence	Assessment tool (cut-off)	Associated factors/Predictors/Risks factors	Protective factors
Country	(observation period)		Internal consistency		
McGinty G., *et al*. (2024) [67] Ireland	N = 1100	11.2% of people met requirements for ICD-11 PTSD (2.4%) or CPTSD (8.8%)	International Trauma Questionnaire (ITQ); response of ≥2 (moderately)	Structural Equation Modeling	N/A
	Age			Number of traumatic events (β = 0.32 to 0.22),	
	Mean = 44.91 ± 15.71				
	Gender		Each ITQ subscale showed satisfactory internal reliability	Loneliness (β = 0.17 to 0.49),	
	M = 528 (48%)			Sleep problems (β = –0.31 to –0.39),	
	F = 569 (51.7%)				
	Other = 3 (0.3%)			Younger age (β = –0.16 to –0.12),	
	These data were collected between 19 March and 9 April 2021, which was a time of strict lockdown measures in the Republic of Ireland.			COVID-19 infection (β = 0.10)	
	Participants recruited by Qualtrics drom existing research panels via email, SMS or in-app notifications.				
Greenblatt-Kimron L., *et al*. (2023) [69] Israel	N = 512	7.4% (38/512)	International Trauma Questionnaire (ITQ) (PTSD: ≥2 in each cluster; CPTSD: PTSD criteria + DSO criteria)	Univariate logistic regression, Multinomial logistic regression Trauma exposure: OR = 1.30, *p* < 0.05; COVID-19 pandemic-related worries: OR = 2.97, *p* < 0.001	N/A
	Age	Of these, 4.1% (21) reported a clinical level of PTSD, while the other 3.3% (17) also reported CPTSD.			
	Range = 68–87				
	mean age 72.67 (SD = 3.81)				
	Gender		Cronbach’s α = 0.897		
	M = 255 (49.8%)				
	F = 257 (50.2%)				
Daly M., *et al*. (2021) [68] Ireland	N = 4193	12.5% in February 2019 (not in COVID-19 pandemic period),	International Trauma Questionnaire (ITQ); presence of one symptom per cluster (score of ≥2) and functional impairment	Binary logistic regression analysis with cluster-robust standard errors	N/A
	1020 respondents in February 2019,				
	COVID-19 pandemic period:			Males (10.8% increase, *p * < 0.001),	
	1041 in April 2020, 1032 in May 2020, 1100 in December 2020; Mean age = 44.5 years, SD = 15.6, 51.5% female, 56.7% had a third-level education	18.0% in April 2020, 22.0% in May 2020, 17.6% in December 2020		Age 18–34 (20.7% to 37.4%),	
			Internal reliabilities were consistently α≥ 0.90 across all time points	No third-level qualification (9.7% to 21.3%),	
				Leinster region (12.8% to 24.5%)	
Makhashvili N., *et al*. (2020) [66] Georgia	N = 2088	PTSD	ITQ	Multivariate regression analyses	Meditation/relaxation exercises (OR 0.39, *p * < 0.01), Physical exercise (OR 0.66, *p* = 0.01), Positive thinking (OR 0.63, *p* = 0.01), Planning for the future (OR 0.59, *p * < 0.01), Reading/TV/radio (OR 0.55, *p* = 0.00), Housework/DIY (OR 0.68, *p* = 0.01)
	Age	F: 11.8%	(score ≥2)	Bad/very bad household economic situation (Coef. 2.66, 95% CI 1.36 to 3.96, *p * < 0.01), Larger household size (Coef. 4.58, 95% CI 2.99 to 6.16, *p * < 0.01), Current NCD (Coef. 1.28, 95% CI 0.08 to 2.49, *p* = 0.04), Symptoms of anxiety (Coef. 5.62, 95% CI 4.45 to 6.79, *p * < 0.01), Adjustment disorder (Coef. 4.57, 95% CI 3.60 to 5.55, *p * < 0.01)	
	Range = 18–70+	M: 12.5%	and adjustment disorder (ADNM8) Good internal reliability with Cronbach’s α ranging from 0.89 to 0.91 across the four measures		
	Gender				
	M = 281 (13.46%)				
	F = 1087 (86.54%)				
	(25 May 2020 and closed on 25 June 2020)				
	Georgian adults recruited through survey weblinks via social and traditional media, key health agencies and investigator networks.				
Shevlin M., *et al*. (2020) [65] UK	N = 2025 Age ≥18 Gender M = 972 (48%) F = 1047 (51.7%) Other = 6 (0.3%) Between 23 and 28 March 2020, UK adults recruited via online platforms	PTSD 16.79 %	ITQ (score ≥2) International Trauma Questionnaire (ITQ); cut-off score: ≥2 (moderately)	Multivariate binary logistic regression to estimate the unique effect of each predictor variable on the likelihood of PTSD.	Multivariate binary logistic regression to estimate the unique effect of each predictor variable on the likelihood of PTSD.
				Age (younger participants): 25–34 years: OR = 1.27, *p* = 0.27 (unadjusted), OR = 0.99, *p* = 0.65 (adjusted); 35–44 years: OR = 1.05, *p* = 0.72 (unadjusted), OR = 0.74, *p* = 0.48 (adjusted); 45–54 years: OR = 0.47, *p* = 0.31 (unadjusted), OR = 0.39, *p* = 0.25 (adjusted); 55–64 years: OR = 0.23, *p* = 0.14 (unadjusted), OR = 0.31, *p* = 0.18 (adjusted); 65+ years: OR = 0.08, *p* = 0.03 (unadjusted), OR = 0.09, *p* = 0.04 (adjusted)	
					Older age: 45–54 years: OR = 0.47, *p* = 0.31 (unadjusted), OR = 0.39, *p* = 0.25 (adjusted); 55–64 years: OR = 0.23, *p* = 0.14 (unadjusted), OR = 0.31, *p* = 0.18 (adjusted); 65+ years: OR = 0.08, *p* = 0.03 (unadjusted), OR = 0.09, *p* = 0.04 (adjusted)
			Cronbach’s α = 0.93		
				Male gender: OR = 1.33, *p* = 0.06 (unadjusted), OR = 1.85, *p * < 0.001 (adjusted)	
				Living in urban area: OR = 3.25, *p * < 0.001 (unadjusted), OR = 1.91, *p* = 0.02 (adjusted)	
					Higher income: £57,930-: OR = 1.24, *p* = 0.82 (unadjusted), OR = 1.27, *p* = 0.82 (adjusted); £38,740-: OR = 1.96, *p* = 0.33 (unadjusted), OR = 1.55, *p* = 0.99 (adjusted); £25,340-: OR = 2.31, *p * < 0.001 (unadjusted), OR = 1.85, *p* = 0.19 (adjusted); £0–15,490: OR = 1.09, *p* = 0.72 (unadjusted), OR = 1.28, *p* = 0.78 (adjusted), OR = 1.27, *p* = 0.97 (adjusted)
				Presence of children: 1 child: OR = 2.68, *p * < 0.001 (unadjusted), OR = 1.83, *p* = 0.02 (adjusted)	
				2 children: OR = 4.17, *p * < 0.001 (unadjusted), OR = 2.56, *p * < 0.001 (adjusted)	
				3+ children: OR = 3.52, *p * < 0.001 (unadjusted), OR = 2.39, *p* = 0.02 (adjusted)	
				Pre-existing health condition (self): OR = 1.25, *p* = 0.92 (unadjusted), OR = 1.21, *p* = 0.83 (adjusted)	
				Pre-existing health condition (someone close): OR = 1.13, *p* = 0.87 (unadjusted), OR = 1.13, *p* = 0.82 (adjusted)	
				COVID-19 infection (self): OR = 2.55, *p* = 0.38 (unadjusted), OR = 1.03, *p* = 0.50 (adjusted)	Lower personal risk perception of COVID-19 infection (1 month): Moderate: OR = 1.92, *p * < 0.001 (unadjusted), OR = 1.88, *p * < 0.001 (adjusted); High: OR = 4.45, *p * < 0.001 (unadjusted), OR = 3.55, *p * < 0.001 (adjusted)
				COVID-19 infection (someone close): OR = 2.39, *p * < 0.001 (unadjusted), OR = 1.70, *p* = 0.04 (adjusted)	
				Perceived risk of COVID-19 infection (1 month): Moderate: OR = 1.92, *p * < 0.001 (unadjusted), OR = 1.88, *p * < 0.001 (adjusted) High: OR = 4.45, *p * < 0.001 (unadjusted), OR = 3.55, *p * < 0.001 (adjusted)	

CPTSD, Complex Post-Traumatic Stress Disorder*; ICD-11, Eleventh Revision of the International Classification of Diseases; ITQ, International Trauma Questionnaire; PTSD, Post-Traumatic Stress Disorder; SMS, Short Message Service; ADNM8, Adjustment Disorder – New Module 8; NCD, neurocognitive disorder; DIY, Do It Yourself. * = (CPTSD) was included in the WHO International Classification of Diseases, 11th Edition.

### Studies Adopting the Primary Care PTSD Screen for DSM-5 (PC-PTSD-5)

In four papers (9.75% of the included studies), the PC-PTSD-5 has been adopted as main assessment tool for evaluating the presence of PTSD, according to a cut-off of 3 [70] or >3 [24, 71, 72]. The prevalence rate of PTSD ranged from 0.5% (at T2 – from 14 January 2021 to 29 March 2021) in the study by Lueger-Schuster *et al*. (2022) [24] carried out in Austria to 25.5% in the study by Généreux *et al*. (2022) [70] carried out in Sweden. Out of four studies using PC-PTSD-5 version, reliability data were available in two studies, with discrete levels of Cronbach’s alpha values, ranging from 0.65 to 0.83 (Table 5, Ref. [24, 70, 71, 72]).

**Table 5.** **Studies using PC-PTSD-5 to assess PTSD**.

Author (year)	Population (observation	PTSD	Assessment tool (cut-off)	Associated factors/Predictors/Risks factors	Protective factors
Country	period)	prevalence	Internal consistency		
Lovik A, *et al*. (2023) [71] Sweden	N = 27,950	8572 (24.5%)	PC-PTSD-5 (4)	Pearson correlation analysis	N/A
	Male: 5171 (18.5%)		Cronbach’s α = 0.77	Lower age (r = –0.1); BMI (r = 0.06); better sleep quality (r = 0.28); lower sleep quantity (r = –0.12); disruption to daily life (r = 0.49); economic difficulties (r = 0.21); higher COVID-19 worries (r = 0.49); number of comorbidities (r = 0.10)	
	Age 18–94 (mean age: 48.7 ± 15.8)				
	(June 2020–June 2021)				
Généreux M, *et al*. (2022) [70] Canada	N = 300	25.5%	PC-PTSD-5 (3 yes out of 5 questions)	N/A	N/A
	Male				
	Age >18		N/A		
	(April 2020)				
Lotzin A., *et al*. (2022) [72] Austria, Croatia, Georgia, Germany, Greece, Italy, Lithuania, Netherlands, Poland, Portugal, Sweden	N = 4607	17.7%	PC-PTSD-5 (score >3)	Logistic regression	Logistic regression
	Gender		PaSS Stressor Subscales	Younger age (OR = 0.77, *p * < 0.001),	Medium income (OR = 0.68, *p* = 0.032),
	M = 1218 (26.4%) F = 3364 (73.0%) Other = 25 (0.5%) Age Range= 18–89 Mean = 43.77 ± 14.38 (from June to November 2020)		N/A	Female gender (OR = 2.07, *p * < 0.001),	High income (OR = 0.62, *p* = 0.012),
				More than 3 hours of daily COVID-19 pandemic-related news consumption (OR = 1.82, *p* = 0.032),	Face-to-face contact less than once a week (OR = 0.65, *p* = 0.011),
				Poor health condition (OR = 2.23, *p * < 0.001),	Face-to-face contact 3–7 times a week (OR = 0.70, *p* = 0.034),
				Current or previous diagnosis of a mental disorder (OR = 4.60, *p * < 0.001),	
					Digital social contact less than once a week (OR = 0.52, *p* = 0.014),
				Trauma exposure during the COVID-19 pandemic (OR = 1.63, *p * < 0.001),	
					Digital social contact 1–7 days a week (OR = 0.44, *p* = 0.001)
				Governmental crisis management and communication (OR = 1.19, *p * < 0.001),	
				Restricted resources (OR = 1.17, *p* = 0.002),	
				Restricted social contact (OR = 1.16, *p* = 0.010),	
				Difficult housing conditions (OR = 1.24, *p * < 0.001)	
Lueger-Schuster B., *et al*. (2022) [24] Austria	N = 234	7.7% (T1) 27 June 2020–22 September 2020,	PC-PTSD-5 (cut-off > 3) ∙ T1 Cronbach’s α = 0.79 ∙ T2 Cronbach’s α = 0.65 ∙ T3 Cronbach’s α = 0.71 ∙ T4 Cronbach’s α = 0.83	Cochran’s Q test, repeated measures ANOVA, two-way mixed ANOVA	N/A
	Gender				
	M = 75 (32.1%)	0.5% (T2) 14 January 2021–29 March 2021,		Higher prevalence of PTSD in females compared to males across all timepoints (Mean difference = 0.59, *p * < 0.01)	
	F = 158 (67.5%)				
	Other = 1 (0.4%)	2.3% (T3) 13 July 2021–8 October 2021,			
	Age	4.5% (T4) 26 November 2021–13 December 2021			
	Range = 21–81				
	Mean = 48.75 ± 15.03				
	T1: 27 June 2020–22 September 2020,				
	T2: 14 January 2021–29 March 2021, 2.3%				
	T3: 13 July 2021–8 October 2021, 4.5%				
	T4: 26 November 2021–13 December 2021				

BMI, Body Mass Index; DSO, disturbances of self-organization; N/A, Not Applicable; OR, Odds Ratio; PaSS, Pandemic Stressor Scale; PTSD, Post-Traumatic Stress Disorder; PC-PTSD-5, Primary Care PTSD Screen for DSM-5; ANOVA, Analysis of Variance.

### Studies Adopting Other Assessment Tools

In the remaining studies (N = 4), other assessment tools have been used, including the Screen for Posttraumatic Stress Symptoms (SPTSS) [73], the Adult Psychiatric Morbidity Survey, the Global Psychotrauma Screen for Post-Traumatic Stress Symptoms (GPS-PTSS) [74] and COVID-19-PTSD Questionnaire (readjusted from the PCL-5) [75], as reported in Table 6 (Ref. [26, 38, 73, 74]). Predictive factors for PTSD were reported in three studies [26, 73, 74], while protective factors were reported in one study only [26]. The population sizes ranged from 84 participants [26] to 18,147 participants [74]. The age range of participants varied from 18-24 years in the study by Gill *et al*. (2022) [26] to 18–89 years in the study by Forte *et al*. (2020) [64]. Prevalence rates ranged from 6% in Gill *et al*. (2022) [26] carried out in Canada, to 37.1% in Rossi *et al*. (2020) [74] carried out in Italy. Studies using other tools to assess PTSD showed mixed reliability results. For example, the COVID-19 PTSD scale demonstrated excellent internal consistency with a Cronbach’s alpha of 0.94, while the Global Psychotrauma Screen for Post-Traumatic Stress Symptoms (GPS-PTSS) subscale reported a lower internal consistency of α = 0.54.

**Table 6.** **Studies using other tools to assess PTSD**.

Author (year)	Population	PTSD prevalence	Assessment tool (cut-off)	Associated factors/Predictors/Risks factors	Protective factors
Country	(observation period)		Internal consistency		
Gill PK, *et al*. (2022) [26] Canada	N = 84	6% (5/84)	Adult Psychiatric Morbidity Survey (at least seven out of nine symptoms related to PTSD - 75% of symptoms) N/A	Logistic Regression	Logistic Regression
	Age Range = 18–24 Gender M = 12 (26%) F = 62 (74%) from 17 June 2020 until 1 July 2020			Family in high-risk setting: OR = 4.30, *p* = 0.013; Reduced income with aid: OR = 2.80, *p* = 0.038;	Essential workers: OR = 0.13, *p* = 0.012
				Daily to hourly social media use for COVID-19 pandemic-related news: OR = 3.24, *p* = 0.020	
Kakaje A, *et al*. (2021) [73] Syria	N = 5588	probable PTSD	Screen for Posttraumatic Stress Symptoms (SPTSS); three or more on avoidance, two or more on arousal, and one or more on re-experience	Forward linear regression, ANOVA,	N/A
	Gender M = 1696 (30.4%) F = 3892 (69.6%) Age Mean = 26.84 ± 7.81 (from 6 April to 13 April 2020)	23.3% (three positive subscales)		Chi-square, *t*-tests Higher PTSD scores in females (*p * < 0.001), distress from war noises (*p * < 0.001), changing place of living due to war (*p * < 0.001), having a chronic medical condition (*p * < 0.001), monthly income adequacy (*p * < 0.001), distress from providing food being affected (R^2^ = 8%, *p * < 0.001), distress from friends or family being infected (R^2^ = 2.6%, *p * < 0.001)	
			SPTSS		
			(cut-off scores:		
			avoidance ≥3;		
			arousal ≥2;		
			reexperience ≥1)		
			K10		
			N/A		
Forte G., *et al*. (2020) [38] Italy	N = 2286	PTSD	COVID-19-PTSD Questionnaire	N/A	N/A
	Age	29.5%	(score >26)		
	Range = 18–74 Mean = 29.61 ± 11.42 Gender M = 580 (25.4%) F = 1706 (74.6%) (March 2020, during the peak of infection and death due to COVID-19 in Italy)		developed starting from the PTSD Check List for DSM-5 (PCL-5)		
			questionnaire (cut-off score of 26)		
			Cronbach’s		
			α = 0.94		
			Cronbach’s alphas were good for the DSM-5 four-factors model (α = 0.70–0.86) and acceptable for the seven-factors model (α = 0.52–0.85).		
Rossi R., *et al*. (2020) [74] Italy	N = 18,147	PTSD	Global Psychotrauma Screen for Post-Traumatic Stress Symptoms (GPS-PTSS) (≥3/5 symptoms)	Seemingly unrelated logistic regression	N/A
	Age	37.14%		Being under quarantine: OR = 1.74, *p * < 0.01;	
	Mean = 38 Gender M = 3653 (20.5%) F = 14,207 (79.5%) 27 March and 6 April 2020		Cronbach’s α = 0.54	COVID-19 pandemic-related stressful event: OR = 1.46, *p * < 0.001;	
				Working activity discontinued: OR = 1.15, *p * < 0.01;	
				Loved one deceased: OR = 1.68, *p * < 0.001;	
				Loved one infected: OR = 1.22, *p * < 0.05	
				Younger age (no OR reported)	

COVID-19, Coronavirus Disease 2019; GPS-PTSS, Global Psychotrauma Screen for Post-Traumatic Stress Symptoms; K10, Kessler Psychological Distress Scale; OR, Odds Ratio; PTSD, Post-Traumatic Stress Disorder; SPTSS, Screen for Posttraumatic Stress Symptoms.

### Risk Factors

The most frequent predictive factor for PTSD was female gender, consistently reported across multiple studies [27, 52, 55, 62, 65, 72].

**Fig. 2.**
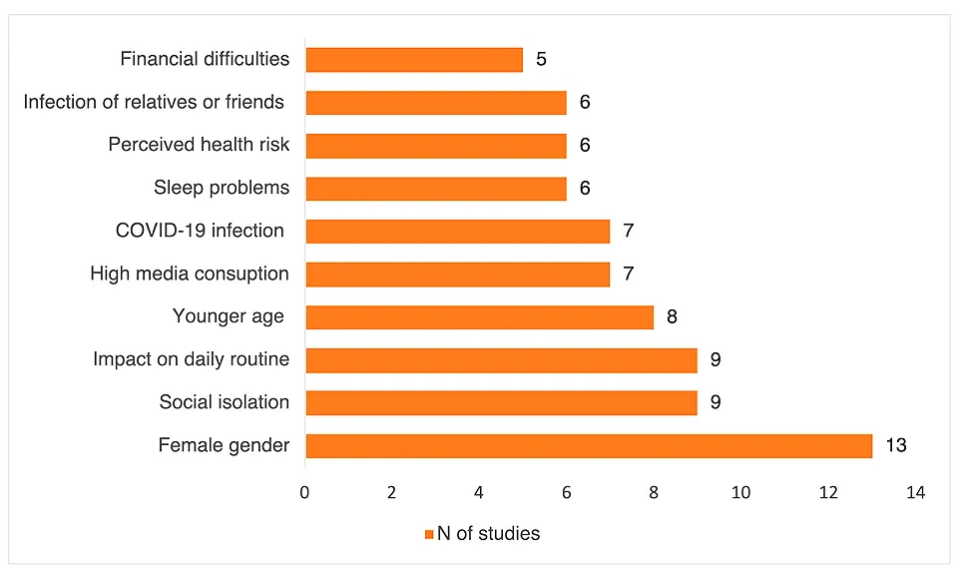
**Graphical summary of the most frequent risk factors of PTSD reported in the included studies**.

Other common predictors of developing PTSD were high COVID-19 pandemic-related stressor score [55], mild to severe anxiety [41], COVID-19 infection [27, 41, 54], recent contact with infected patients [27], family member or loved ones being infected with COVID-19 [27], low levels of self-efficacy, lack of social support [51], negative coping styles [51], financial difficulties (e.g., Odds Ratio (OR) = 1.51, *p* < 0.001) [27] and difficult housing conditions [72], high media consumption of COVID-19 pandemic-related information (e.g., Relative Risk (RR) = 1.53, *p* < 0.001) [41] and perceived health risk of the disease [43] (Fig. 2).

### Protective Factors

Several protective factors for PTSD were identified, including higher gratitude (e.g., β = –0.13, 95%) [56], older age (e.g., OR = 0.48) [44, 62], social support (e.g., β = –0.19, *p* = 0.002) [40], high levels of resilience (e.g., OR = 0.58, *p* < 0.05) [54], and high levels of health literacy [53].

Other factors protecting from the risk of developing PTSD included medium to high income [72], face-to-face contact less than once a week [72], face-to-face contact 3-7 times a week [72], digital social contact less than once a week [72], being an healthcare provider [56], not living with close relatives [62], and working from home [62].

**Fig. 3.**
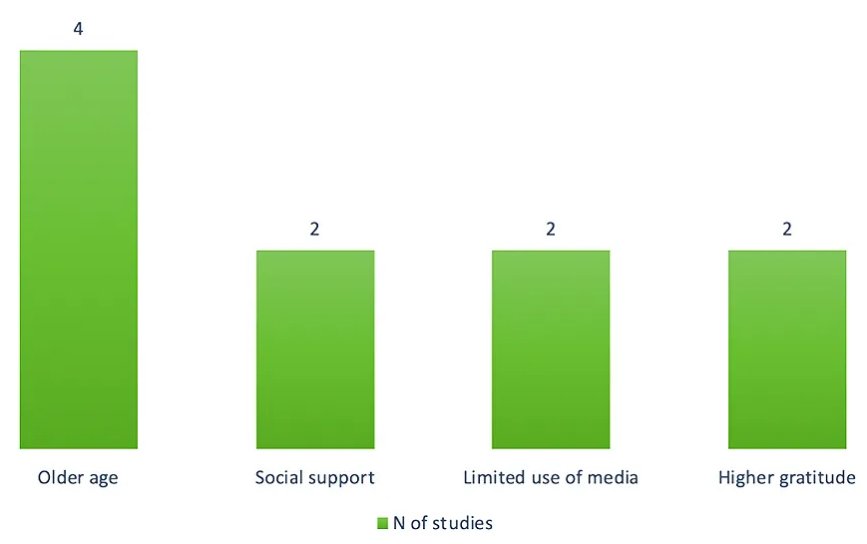
**Graphical summary of the most frequent protective factors of PTSD reported in the included studies**.

Studies by Makhashvili *et al*. (2020) [66] and Shevlin *et al*. (2020) [65] identified as further protective factors the following elements: low levels of personal risk perception of COVID-19 infection, meditation/relaxation exercises, practicing physical exercise, positive thinking, planning for the future, reading/TV/radio, and doing housework (Fig. 3).

## Discussion

The COVID-19 pandemic has been a very stressful event with a detrimental impact on the mental health of the general population [76, 77, 78, 79, 80]. In particular, the COVID-19 pandemic has been considered a “trauma” since it has been associated with high levels of mortality and morbidity as well as with a severe disruption of ordinary activity in daily life [81, 82, 83, 84]. In particular, the severe insecurity of a contagious life-threating virus, the fear of being infected or being hospitalized, the loss of a loved one, and mandatory containment measures have been perceived as traumatic events by the general population, especially by those with inadequate coping strategies. Moreover, the prolonged psychological impact of COVID-19 pandemic, especially in individuals experiencing long-COVID syndrome, may play a substantial role in the development and persistence of chronic PTSD symptoms. This condition is often accompanied by significant neuropsychiatric manifestations, such as heightened levels of anxiety, persistent depression, and notable cognitive impairments, all of which can exacerbate or sustain PTSD over time [79].

However, some other authors have pointed out that the COVID-19 pandemic per se should not be considered a “trauma” for most people, as the term “trauma” usually defines actual or threatened death or serious injury [85]. It should be that the COVID-19 pandemic experience varied greatly among individuals, with someone facing life-threatening illness or loss of close ones, whereas others were less impacted from the consequences of the COVID-19 pandemic and its containment measures [86, 87]. It is of extreme interest to evaluate the long-term consequences of the COVID-19 pandemic on mental health. Most available data have been focused on the immediate aftermath of the worldwide health, economic and social emergency, but some experts have warned that the “long wave” of COVID-19 pandemic will be observed in the next decades. It could be that the perceived insecurity, hopelessness and drastic change in ordinary life due to the acute phase of the pandemic have left some “scars” in the general population, that will be understood time by time, since these are time-consuming processes. Moreover, a long-term consequence of the COVID-19 pandemic could be the development of a chronic form of PTSD, which is a longer-lasting form of the disorder occurring when symptoms last for more than three months. People with chronic PTSD may have more severe and persistent symptoms, including difficulty with relationships, work, and daily activities. Specific strategies to identify people at high-risk for developing chronic PTSD should be developed in order to deliver tailored supportive and preventive interventions.

Several studies have found an unprecedented increase in mental health problems during the COVID-19 pandemic and in its aftermath, particularly in terms of anxiety and depressive disorders. Moreover, the outbreak of the health crisis and its containment measures have represented stressful experiences occurred in a very short period, which have been associated with a high rate of stress-related disorders [17, 88, 89, 90, 91].

Previous outbreaks of infectious disease (e.g., SARS, Ebola, and Middle East Respiratory Syndrome (MERS)) have shown the detrimental influences of disease-related stress on emerging acute distress [92, 93, 94]. Similarly, the COVID-19 pandemic itself and lockdown measures can also induce similar problems in the population involved. However, recent studies showed heterogeneity in the COVID-19 pandemic response depending on individual characteristics and area-specific factors [3, 95, 96, 97, 98, 99, 100, 101, 102, 103, 104, 105]. These data are consistent with those found in other emergency situations; for example, in earthquakes, a correlation has been found between the degree of psychopathology and the distance from the epicenter and, therefore, the degree of exposure to the event [106, 107].

Several authors have investigated PTSD prevalence rates during the COVID-19 pandemic across various countries. Many of these researchers have also studied the risk and protective factors associated, that could be helpful for management of PTSD. To our knowledge, this is the first review to examine PTSD prevalence rates in different countries during the various waves of COVID-19 pandemic, focusing on both predictive and protective factors. The main findings of the present systematic review are the following: (1) a significant heterogeneity in prevalence rate of PTSD; (2) extreme variance in threshold value considered by the different research studies; (3) PTSD prevalence rates in the general population during the COVID-19 pandemic are higher compared to estimates obtained during the previous decade [108].

A significant finding is that the majority of studies included in this research were conducted in Italy and China, representing approximately 19.5% of the total studies included. The prominence of these two countries in the dataset may be attributed to the fact that they were the first countries to be heavily affected by the COVID-19 pandemic, potentially causing greater distress in the local populations, compared to those that had more time to adopt restrictive measures and adapt to their consequences.

A clear finding from our review is the diversity in prevalence data across the various selected studies. This variability may be attributed to several factors. One reason could be the use of different tools, each characterized by specific psychometric properties. It must be observed that such clinical heterogeneity, stemming from the diverse contexts of data collection in the included observational studies, limits the generalizability of the results. However, according to our results, it is possible to state that the highest prevalence rates were observed in the Passavanti *et al*. [25] (2021) study using the IES-R, while the lowest rates were reported by Lueger-Schuster *et al*. [24], who adopted PC-PTSD-5. This finding could be attributed to the different sensitivity of the adopted assessment tools, highlighting the importance of selecting reliable evaluation instruments when conducting prevalence studies. Another explanation could lie in the geographical contexts in which the assessments were carried out. Indeed, COVID-19 pandemic waves occurred at different times in different areas, which may have been prepared differently for the emergency. The fact that some countries were not prepared to manage the consequences of a pandemic, unlike others that had prepared for the emergency in the meantime, might have influenced the individual perception of the event as traumatic. Studies using the IES-R reported higher prevalence rates compared to those employing other assessment tools. The study by Passavanti *et al*. [25] (2021), which employed the IES-R, reported the highest prevalence rate (70.16%). This may suggest that the IES-R may be more sensitive in detecting PTSD symptoms, although it could also reflect differences in the cut-off criteria adopted among the different scales.

A significant aspect emerging from our research is the variety of the assessment tools used. Versions of PCL were the most utilized tools, employed in 17 studies, accounting for approximately 41.5% of the total studies. The IES-R was used in 11 studies (26.8%), the ITQ in 5 studies (12.2%), the PC-PTSD-5 in 4 studies (9.7%), and other tools were used in 4 studies (9.7%). Such heterogeneity indicates that there is no consensus on the most suitable self-reported tool for diagnosing PTSD in the general population, although the Clinician-Administered PTSD Scale for DSM-5 (CAPS-5) [29] is considered the gold standard [109]. Furthermore, the same scale can exist in different forms, such as the PCL-5. This suggests the need for a standardized tool for PTSD assessment. All scales used primarily assess the severity of PTSD-related symptoms rather than providing a definitive diagnosis. This highlights the necessity for further research to develop assessment tools that can deliver a definitive PTSD diagnosis. Furthermore, significant differences in cut-off scores among the same scales, such as the IES and PCL, indicate variability in assessment criteria across different studies, which could impact the comparability of PTSD prevalence rates and associated risk factors. Thus, standardizing cut-off scores and assessment criteria could enhance the reliability and validity of PTSD research outcomes.

Another source of heterogeneity is represented by the extreme variation in sample sizes among the included studies, ranging from 84 participants in the study by Gill *et al*. (2022) [26] to 31,557 participants in the study by Elhadi *et al*. (2022) [27]. This variability underscores the diverse methodological approaches adopted by researchers in different contexts. This heterogeneity in sample size makes it very complicated to compare results among studies, which goes to compromise generalizability. Since our review focuses on observational studies, a statistical heterogeneity analysis was not applicable.

Such a lack of consistent data on the prevalence of post-traumatic stress disorder prevents proper health planning and resource allocation for mental health services [110, 111]. It also limits the development of targeted interventions, as the diverse experiences of different populations may be inaccurately reflected [112, 113, 114]. Other decisions based on such heterogeneous data could result in ineffective health policies [90]. In addition, the variability in reported prevalence has some implications with regard to how the public views and recognizes PTSD as a real mental disorder. To overcome these challenges, future studies need to include more narrow and standardized methodological processes.

Several risk factors for developing PTSD have been identified [115]. Female gender emerged as the most important predictive factor, consistently reported across multiple studies [27, 72]. This finding is in line with existing literature suggesting that females are more susceptible to PTSD [21], possibly due to biological, psychological, and social factors. Younger age was another significant risk factor, reported with significant odds ratios [72]. The increased vulnerability of younger individuals to PTSD could be attributed to less developed coping mechanisms and higher exposure to stressors. COVID-19 infection itself was a significant risk factor [54], highlighting the direct psychological impact of the disease. Financial issues were also frequently reported [27], reflecting the economic strain imposed by the COVID-19 pandemic. Additionally, high media consumption of COVID-19 pandemic related information emerged as a significant risk factor [41], suggesting that constant exposure to distressing news can exacerbate psychological distress.

Several protective factors were identified as well. High resilience was the most significant protective factor, consistently reported across studies. Resilience, characterized by the ability to adapt and recover from adversity, plays a crucial role in mitigating the impact of traumatic events. Higher gratitude was reported as a significant protective factor, suggesting that a positive outlook and appreciation for life can buffer against PTSD. Older age was also identified as a protective factor [62], possibly due to more developed coping strategies and life experience. Social support was another significant protective factor [40]. The presence of a supportive network can provide emotional comfort and practical assistance, reducing the likelihood of developing PTSD.

The present study has some limitations that must be acknowledged. Firstly, the focus was on the general population, which may have led to an underestimation of prevalence rates compared to specific groups, such as healthcare professionals working on the frontline during the COVID-19 pandemic.

In the included studies, PTSD was largely assessed by means of standardized but not COVID-19 pandemic-specific instruments. For this reason, the current results may have been affected by confounding biases due to independent traumatic factors occurred in the meantime.

Similarly, the mechanisms by which predictive factors lead to PTSD cannot be easily explained by our findings, due to the varying extent of detrimental exposures (e.g., loss of a loved one due to the COVID-19, temporary disruption of daily routine, work habits changes, etc.) and the independent relationship between several risk factors and the COVID-19 pandemic (e.g., gender female, lower socioeconomic status).

The reliability analysis of the included studies was conducted based on the data available in the individual studies. Overall, the majority of tools demonstrated good to excellent internal consistency, as evidenced by Cronbach’s alpha values generally above 0.85. However, it is important to note that reliability data were not available for all studies, thus limiting the scope of the analysis. For studies lacking this information, it was not possible to evaluate their reliability, and estimations could not be made. Despite these limitations, the findings underscore the robust psychometric properties of the tools used to assess PTSD across different cultural and linguistic contexts.

The included studies were carried out in different periods across 2020 and 2021, reflecting diverse stages of the COVID-19 pandemic. Indeed, the first months of the emergency differed from the following year, when vaccinations became available. In this regard, a study carried out in Bangladesh by Alam *et al*. [116] highlighted lower prevalence of PTSD symptoms among vaccinated people compared to unvaccinated ones.

Another significant limitation is the inclusion of only studies published in English. This criterion may have excluded numerous studies in other languages, particularly Asian languages, that could contain valuable data.

Furthermore, a meta-analysis was not conducted due to the heterogeneity of the data. The variability in study designs, sample sizes, and assessment tools limited the possibility of performing a meta-analysis.

This study highlights the diverse methodological approaches and variability in assessment criteria across different studies on PTSD during the COVID-19 pandemic. The identification of consistent risk and protective factors provides valuable insights for targeted interventions and support strategies. Future research should aim to standardize assessment tools and criteria to enhance the comparability and reliability of findings in PTSD research.

## Conclusions

COVID-19 pandemic has resulted in detrimental effects on mental health of special groups of people exposed to contagion (e.g., infected patients, healthcare workers, etc.) as well as the general population, who experienced disruption of daily routine and the adoption of new habits. Therefore, many studies have investigated the PTSD effects on the general population across the world.

The current systematic review focused on prevalence rates, psychometric tool used, predictors and protective factors of PTSD as they were found in general population-based observational studies performed during the COVID-19 pandemic. Heterogeneity due to different instruments and diverse cut-offs was highlighted across the literature included. Moreover, the identification of consistent risk and protective factors provides valuable insights for targeted interventions and support strategies. Future research should aim to standardize assessment tools and criteria to enhance the comparability and reliability of findings in PTSD research.

## Availability of Data and Materials

All data generated or analyzed during this study are included in this published article.

## Supplementary Material

Supplementary material associated with this article can be found, in the online version, at https://doi.org/10.62641/aep.v53i4.1882.
